# Erdheim-Chester disease: unveiling hidden manifestations through two case reports

**DOI:** 10.3389/fonc.2025.1611907

**Published:** 2025-09-22

**Authors:** Guoxia Zhang, Xiaoqing Yang, Lei Fang, Yuxia Cheng, Yanhua Duan, Jing Cui

**Affiliations:** ^1^ Department of Pathology, The First Affiliated Hospital of Shandong First Medical University & Shandong Provincial Qianfoshan Hospital, Jinan, Shangdong, China; ^2^ Department of Nuclear Medicine, The First Affiliated Hospital of Shandong First Medical University & Shandong Provincial Qianfoshan Hospital, Jinan, SD, China

**Keywords:** Erdheim-Chester disease, histiocytosis, endometrium, pericardium, BRAF V600E, differential diagnosis

## Abstract

We present two cases of Erdheim-Chester disease (ECD) in 66-year-old female patients, highlighting their distinct clinical manifestations and diagnostic challenges. The first patient presented with chest tightness and dyspnea, revealing pericardial effusion and hypermetabolic foci in multiple organs on imaging. Histopathological analysis indicated abundant foamy histiocytes, confirming ECD. Significant reduction in metabolic lesions following cladribine treatment. The second patient developed subcutaneous nodules post-hysterectomy, later diagnosed with mixed lobular panniculitis. Subsequent biopsies revealed similar histiocyte profiles, leading to ECD diagnosis. She responded well to an immunomodulatory regimen. Notably, whole-exome sequencing detected a *MAP2K1* mutation in the second case, ​which lacked​ the BRAF V600E mutation commonly associated with ECD. These cases underscore the importance of thorough diagnostic evaluation and highlight the variability in clinical presentation and genetic alterations of ECD, contributing to improved recognition and management strategies for this rare condition.

## Introduction

Erdheim-Chester disease (ECD) is a rare form of non-Langerhans cell histiocytosis characterized by the infiltration of foamy histiocytes into various tissues and resulting in a wide spectrum of clinical manifestations (1, 2). This disease predominantly affects adults and is associated with a diverse range of symptoms including bone pain, cardiovascular complications, and neurological deficits, among others (3). The diagnosis of ECD is often challenging due to its heterogeneous presentation and the requirement for histopathological confirmation, which typically involves the identification of CD68-positive, CD1a-negative histiocytes in tissue biopsies (4, 5). Imaging studies, such as positron emission tomography-computed tomography (PET-CT) play a crucial role in revealing hypermetabolic lesions in affected organs and thus aid in the diagnosis of ECD (6, 7).

Various therapeutic approaches have been explored, including chemotherapy and targeted therapies; however, the optimal management strategy for ECD remains to be fully established. The presence of *BRAF* mutations, particularly the V600E variant, has been identified in some ECD patients, thereby complicating the genetic landscape of the disease (8–10).

In this report, we present two cases of ECD in 66-year-old female patients, emphasizing the diverse clinical presentations and diagnostic challenges. The first case involved a patient with pericardial effusion and systemic involvement. The second case demonstrated an insidious progression of endometrial involvement, which subsequently advanced to manifest in cutaneous, postoperative vaginal stump, and bladder neck regions. The diagnostic process was marked by a complex clinical trajectory.

## Case presentation

### Case 1

**Figure 1:**
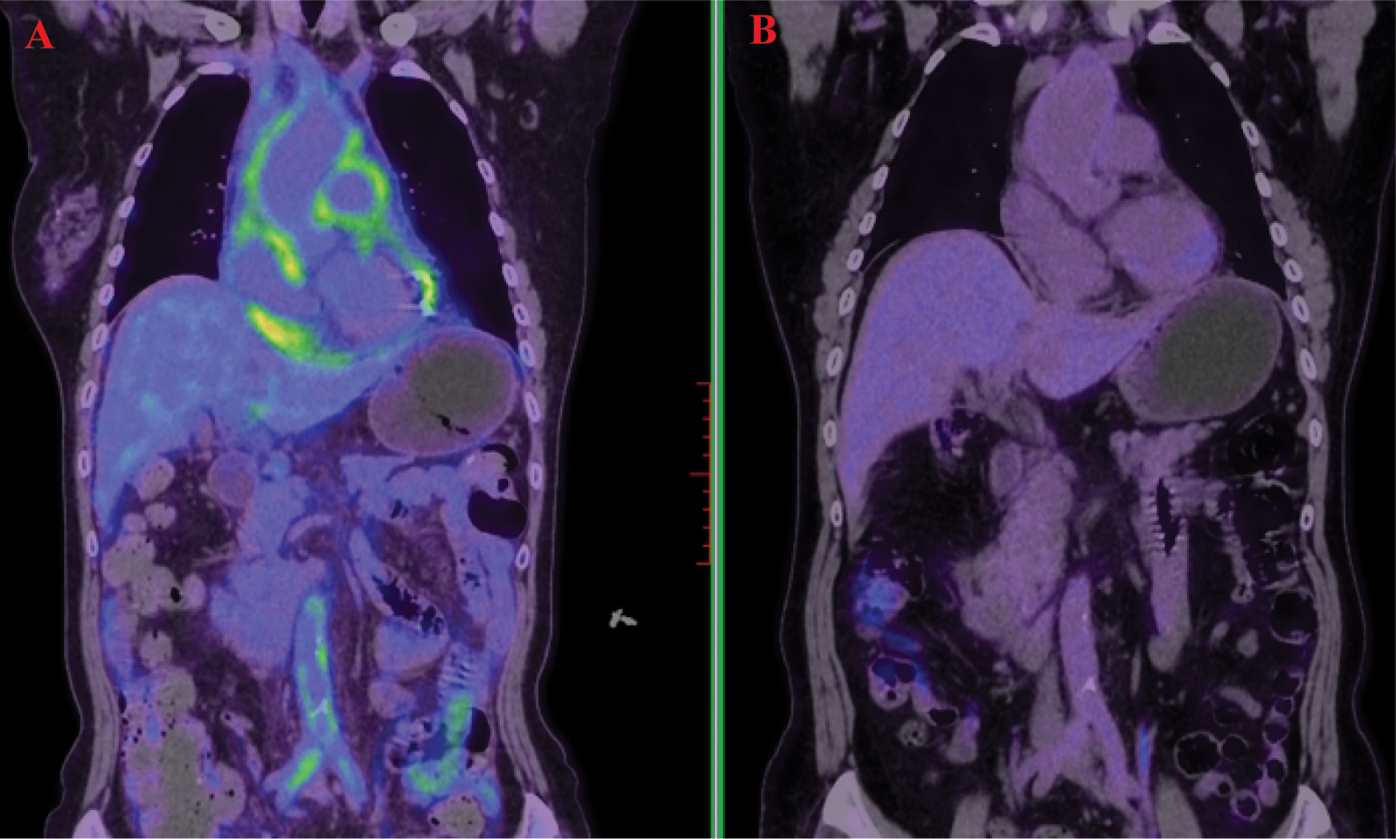
18F-Fluorodeoxyglucose positron emission tomography/computed tomography (FDG-PET/CT) demonstrated high uptake in the pericardium, aorta, abdominal aorta, and bilateral iliac artery walls **(A)**. A follow-up scan after treatment showed marked metabolic reduction in these previously uptake regions **(B)**.

**Figure 2:**
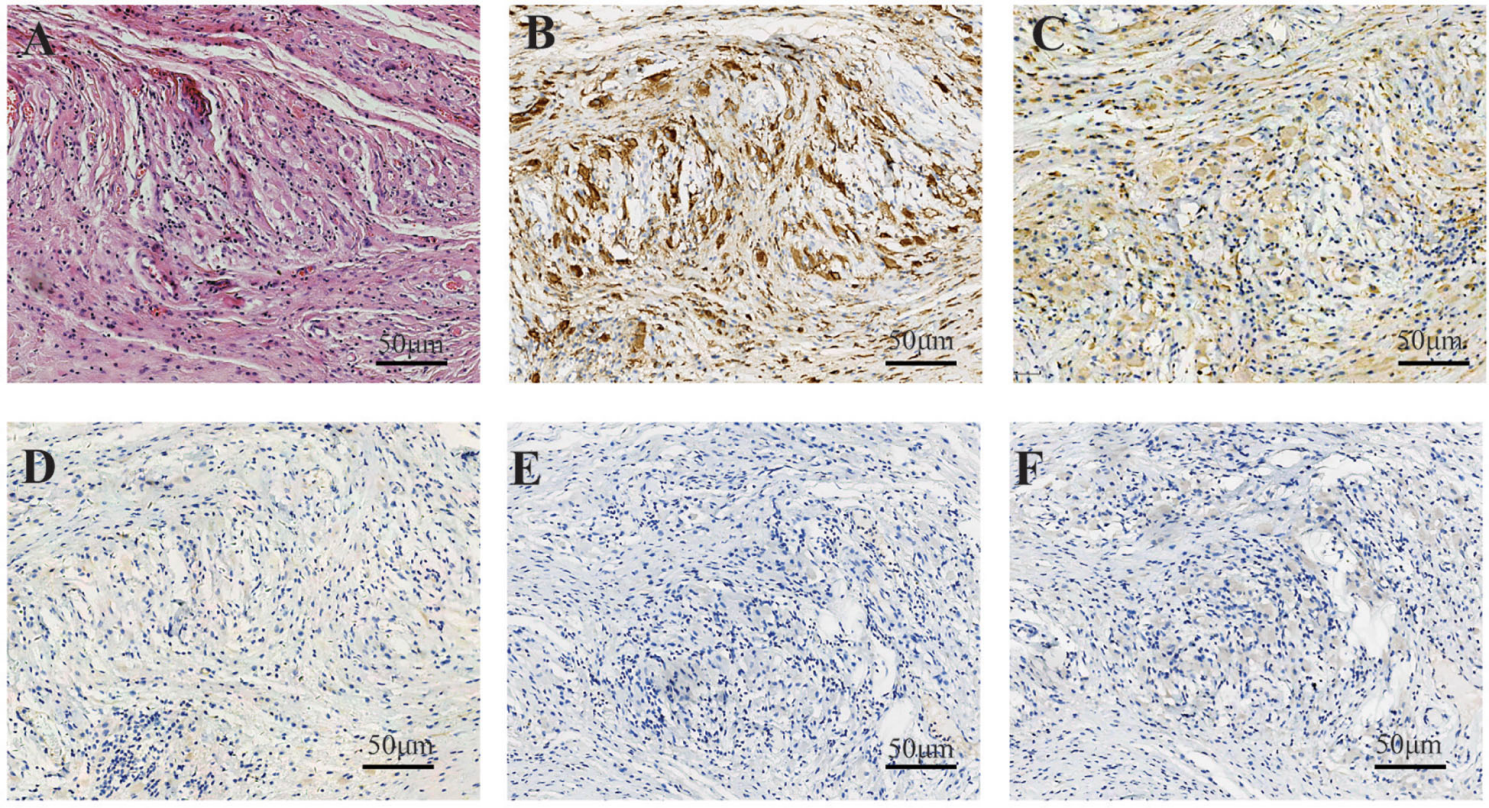
In case 1, the fibrous tissue demonstrated an infiltrate of bland-appearing histiocytes characterized by abundant foamy cytoplasm, accompanied by scattered lymphocytes **(A)**. Immunohistochemical staining showed strong positive cytoplasmic staining for CD168 in histiocytes **(B)** and moderate staining for CD38 **(C)**. Immunohistochemical staining showed that the tissue cells were negative for CD1a **(D)**, Langerin **(E)**, and BRAF **(F)**.

A 66-year-old female patient was admitted on November 3, 2022, with chief complaints of chest tightness and dyspnea. Cardiac magnetic resonance imaging (MRI) revealed extensive thickening of the pericardium and irregularly shaped tissue that had infiltrated the wall of the right atrium, also impacting the interatrial septum. The 18F-Fluorodeoxyglucose positron emission tomography/computed tomography (18F-FDG PET-CT) exhibited significant uptake in the right atrial wall, interatrial septum, pericardium, pulmonary artery, aorta, and vena cava (**Figure 1A**). The patient subsequently underwent pericardial window surgery and biopsy, during which pericardial adhesions and nodular changes in the epicardial fat were observed. Histopathological examination showed abundant foamy histiocytes and multinucleated giant cells within the pericardium and surrounding adipose tissue. Immunohistochemistry revealed CD163(+) and CD68(+) expression in foamy cells, while Langerin, CD1a, and BRAF were negative (**Figure 2**). These findings led to a diagnosis of Erdheim-Chester disease (ECD).

The patient received ​combination therapy​ with subcutaneous interferon-α (3 million IU three times weekly) and cladribine (4 cycles of 7-day infusions at 4-week intervals). ​A significant reduction in metabolic lesions​ was observed following combination therapy (**Figure 1B**). Interferon-α monotherapy (3 million IU subcutaneously weekly) was continued as ​maintenance therapy for 12 months, with ​disease surveillance via MRI every 6 months. No grade ≥2 adverse events occurred during this phase. Follow-up involved cardiac ultrasonography and lab tests including CBC, liver/renal function, CRP, ESR, IL-6, and BNP. To date, the patient remains in stable condition with no disease progression.

### Case 2

A 66-year-old female patient underwent hysterectomy with bilateral adnexectomy for “leiomyoma of uterus” at another hospital in March 2023. Histopathological analysis revealed a well-circumscribed uterine leiomyoma within the myometrium, accompanied by the presence of lipid-laden histiocytes infiltrated in the endometrial stroma. In May 2023, the patient developed multiple subcutaneous nodules with partial ulceration. On August 14, 2023, the patient was admitted to the Shandong Provincial Qianfoshan Hospital. A skin nodule biopsy showed clear cytoplasmic “lipid-like” cells and lymphocytic infiltration under the microscope, leading to a diagnosis of mixed lobular panniculitis. The patient was treated with prednisone and cyclophosphamide, resulting in significant healing of skin ulcers and reduction of nodules within three months.

**Figure 3:**
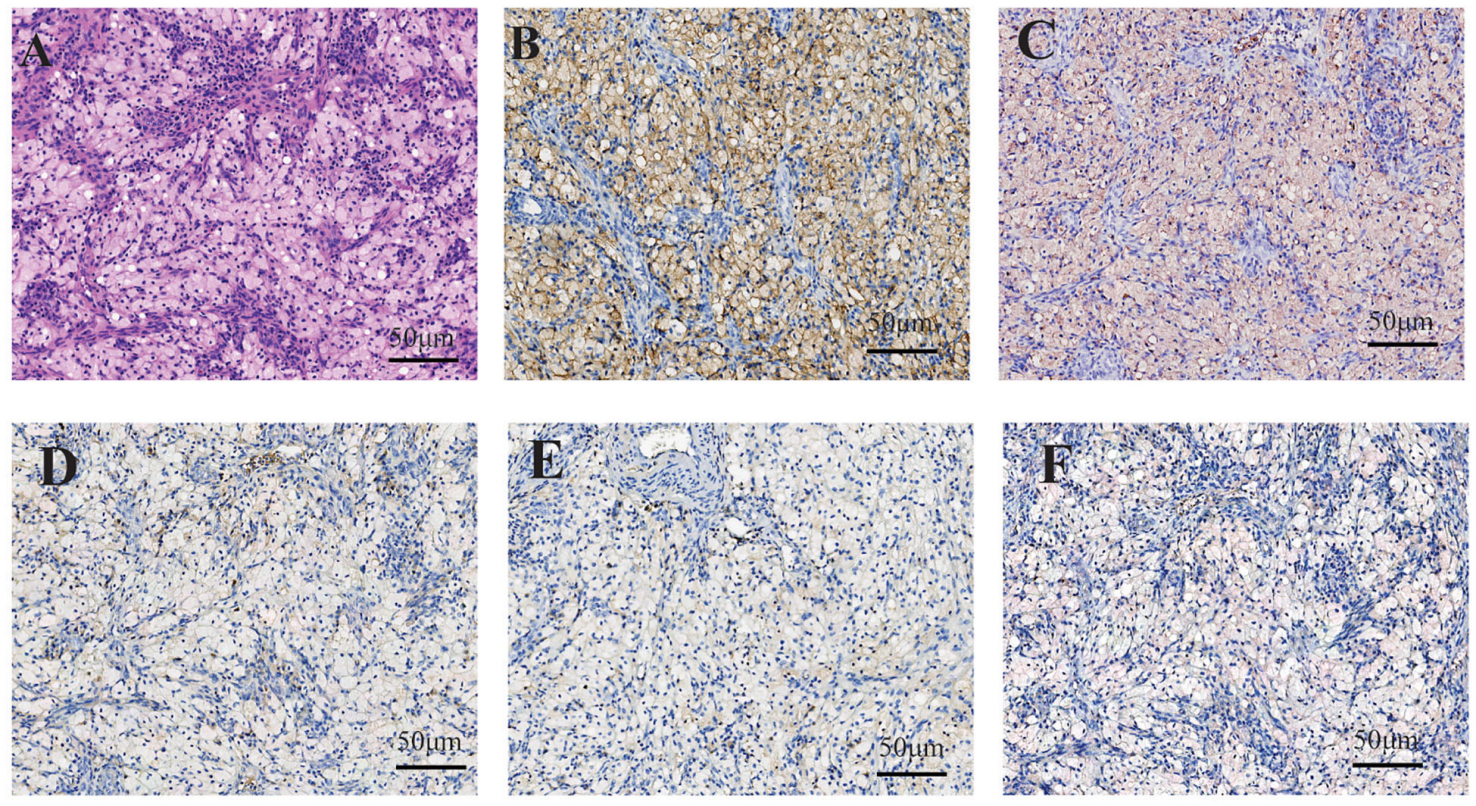
In case 2, within regions of histiocyte aggregation, hyperplasia of small blood vessels and scattered lymphocytic infiltration were observed. The histiocytes exhibited clear cytoplasm, small nuclei, and distinct cell boundaries **(A)**. Immunohistochemical staining demonstrated cytoplasmic positivity for CD168 **(B)** and CD38 **(C)** in histiocytes, whereas staining for CD1a **(D)**, Langerin **(E)** and BRAF **(F)** were negative.

In November 2023, the patient presented with vaginal stump bleeding and bladder neck obstruction and was readmitted. A biopsy of the vaginal stump revealed dense aggregates of histiocytes in the fibrous tissue. Immunohistochemical staining showed histiocytes positive for CD68 and CD163, but negative for Langerin, CD1a, and BRAF (**Figure 3**).

Re-examination of the initial endometrial specimen and the skin biopsy revealed similar immunohistochemical profiles in the previously identified so-called “clear cells,” confirming CD68(+) and CD163(+) expression with negative markers for ECD.

Genomic DNA was extracted from formalin-fixed, paraffin-embedded (FFPE) specimens, specifically pericardial biopsy tissue from patient 1 and vaginal cuff tissue from patient 2, utilizing a standardized protocol. These DNA samples were subsequently ​analyzed by​ whole-exome sequencing (WES) using next-generation sequencing (NGS) technology. The results revealed that neither patient harbored the *BRAF V600E* mutation. However, *MAP2K1* mutation was identified in Case 2, with no additional mutations detected in other common driver genes, including *ARAF*, *NRAS*, *KRAS*, or *PIK3CA*.

Given the identification of a *MAP2K1* mutation, the patient was initiated on a therapeutic regimen of trametinib in combination with pegylated interferon-alpha. Following significant clinical improvement, the patient transitioned to routine outpatient follow-up, which included serial imaging and laboratory monitoring. The disease remains stable without evidence of progression. However, persistent urinary incontinence is noted as a sequela of prior bladder outlet obstruction that was treated with bladder-neck dilation.

## Discussion

The cases presented herein underscore the complexity and variability of Erdheim-Chester disease (ECD) manifestations, highlighting the necessity for clinical vigilance in both diagnosis and management. The first case illustrates a patient with progressive dyspnea, orthopnea, and atypical chest pain. In this instance, PET/CT imaging was instrumental in guiding the diagnostic process, which was ultimately confirmed through pericardial cytology. The second case, however, highlights the diagnostic challenges that can arise in atypical presentations. A retrospective pathological review revealed that the initial pathological assessment of the uterine leiomyoma resection and subsequent skin biopsy failed to recognize histiocytic proliferation, leading to a pathological diagnosis that diverged from the correct clinical diagnosis. Collectively, these cases expand the recognized spectrum of ECD and reinforce the importance of maintaining a high index of suspicion, particularly in the context of atypical clinical scenarios.

Erdheim–Chester disease (ECD) is a rare, multi-systemic histiocytosis driven by MAPK-pathway activation. Treatment stratification is crucial: BRAF V600E mutation carriers receive BRAF inhibitors (e.g., vemurafenib) as first-line therapy, achieving 43–88% response rates and significant CNS/cardiac improvement. BRAF-wild-type cases are primarily treated with interferon-alpha (IFN-α), increasing 5-year survival to 80%. Refractory cases may benefit from IL-1 blockade (anakinra), chemotherapy (cladribine), or mTOR inhibition (sirolimus). Efficacy assessment integrates imaging (PET-CT for metabolic activity, cardiac MRI for infiltration/effusion), clinical manifestations (bone pain, exophthalmos, exercise tolerance), and laboratory markers (CRP, ESR, IL-6, and organ-function parameters like eGFR).

Histopathological evaluation of lesion biopsy remains the cornerstone of ECD diagnosis and exclusion of differential diagnoses (5, 11). Histological examination typically reveals characteristic lipid-laden histiocytic infiltrates with fibro-inflammatory stromal changes, often demonstrating multinucleated Touton giant cells (5, 12). Immunohistochemical profiling exhibits distinct patterns: ECD histiocytes consistently express CD68 and CD163, while demonstrating absence of CD1a and langerin (13, 14).

From a pathological perspective, ECD must be differentiated from several other conditions: (1) Langerhans Cell Histiocytosis (LCH): Histopathologically, LCH is marked by mature Langerhans cells with reniform or coffee-bean nuclei, nuclear grooves, and perivascular eosinophilic infiltration. ECD, in contrast, lacks nuclear grooves and exhibits CD1a/S100 negativity, distinguishing it from LCH, which demonstrates CD1a/S100 positivity (15, 16).(2) Juvenile Xanthogranuloma (JXG): JXG is characterized by dermal infiltration of foam cells, Touton multinucleated giant cells, and mild lymphocytic infiltration. While immunophenotypic overlap exists, JXG predominantly affects infants and lacks osseous sclerosis or perivascular infiltration. ECD, however, is associated with systemic multi-organ involvement, particularly affecting internal organs (17, 18). (3) Rosai-Dorfman Disease (RDD): RDD is defined by sinusoidal histiocytic proliferation with emperipolesis (histiocytes engulfing lymphocytes/plasma cells) and prominent plasma cell infiltration. RDD exhibits S100 positivity, whereas ECD lacks both emperipolesis and S100 expression (19–21). (4) Panniculitis: Panniculitis presents as interstitial adipose tissue inflammation with lymphocytes, plasma cells, neutrophils, and lipid-laden macrophages causing cytoplasmic vacuolization. Overlapping morphological features may lead to misdiagnosis of ECD as panniculitis, particularly when lipid-laden histiocytic infiltration is underrecognized. This diagnostic pitfall is exemplified in the second case, where prior pathologists overlooked the lipid-laden histiocytic component, resulting in erroneous conclusions.

In the diagnostic workup of histiocytic neoplasms, genetic alteration analysis, particularly *BRAF* molecular profiling, serves as a cornerstone for distinguishing Erdheim–Chester disease (ECD) from other entities (22, 23). Nevertheless, the presence of BRAF V600E mutations is not exclusive to ECD, as these have also been documented in other histiocytosis, such as Langerhans cell histiocytosis (LCH) and juvenile xanthogranuloma (JXG). In such instances, a meticulous morphologic evaluation coupled with a comprehensive immunohistochemical panel becomes indispensable for accurate classification. Conversely, Rosai–Dorfman disease (RDD) exhibits an extremely low incidence of *BRAF* alterations, a notable disparity that significantly facilitates its differentiation from ECD.

## Conclusion

In this report, we describe two cases of Erdheim-Chester disease (ECD) without skeletal involvement. Notably, we present an exceptionally rare case of ECD manifesting as covert endometrial involvement as the initial clinical presentation.

## Data Availability

The raw sequencing data supporting the findings of this study have been deposited in the NCBI Sequence Read Archive (SRA) under the BioProject accession number PRJNA1328182. These data are publicly available and can be accessed through the NCBI SRA database https://www.ncbi.nlm.nih.gov/bioproject/PRJNA1328182.
